# The Existence of Weak *𝒟*-Pullback Exponential Attractor for Nonautonomous Dynamical System

**DOI:** 10.1155/2016/1871602

**Published:** 2016-03-22

**Authors:** Yongjun Li, Xiaona Wei, Yanhong Zhang

**Affiliations:** School of Mathematics, Lanzhou City University, Lanzhou 730070, China

## Abstract

First, for a process {*U*(*t*, *τ*)∣*t* ≥ *τ*}, we introduce a new concept, called the weak *𝒟*-pullback exponential attractor, which is a family of sets {*ℳ*(*t*)∣*t* ≤ *T*}, for any *T* ∈ *ℝ*, satisfying the following: (i) *ℳ*(*t*) is compact, (ii) *ℳ*(*t*) is positively invariant, that is, *U*(*t*, *τ*)*ℳ*(*τ*) ⊂ *ℳ*(*t*), and (iii) there exist *k*, *l* > 0 such that dist(*U*(*t*, *τ*)*B*(*τ*), *ℳ*(*t*)) ≤ *ke* ^−(*t*−*τ*)^; that is, *ℳ*(*t*) pullback exponential attracts *B*(*τ*). Then we give a method to obtain the existence of weak *𝒟*-pullback exponential attractors for a process. As an application, we obtain the existence of weak *𝒟*-pullback exponential attractor for reaction diffusion equation in *H*_0_^1^ with exponential growth of the external force.

## 1. Introduction

Pullback attractor is a suitable concept to describe the long time behavior of infinite dimensional nonautonomous dynamical systems or process generated by nonautonomous partial differential equations. There are many references concerned with the existence of pullback attractors for nonautonomous PDEs (see [1–5]). In [2], Caraballo introduced the notion of *𝒟*-pullback attractor for nonautonomous dynamical systems and gave a general method to prove the existence of *𝒟*-pullback attractor. However, pullback attractors or *𝒟*-pullback attractors attract any bounded set of phase space, but the attraction to it may be arbitrarily slow. In order to describe the attracting speed, the concept of pullback exponential attractor is put forward (see [6]), which is a positively invariant family of compact subsets with finite fractal dimension (see [7, 8]) and exponentially attracts each bounded subset. In [6], a new method is given to prove the existence of pullback exponential attractor and it is applied to reaction diffusion equation when the external force is normal; in [9], the same result is obtained when the nonlinear term *f*(*t*, *u*) satisfies |*f*(*t*, *u*) − *f*(*t*, *v*)| ≤ *ξ*(*t*)|*u* − *v*|. In fact, these conditions are relatively strict; for general conditions, we can not get the result.

Motivated by these problems and some ideas in [3–6], we introduce a new attractor, called the weak *𝒟*-pullback exponential attractors that is for a process {*U*(*t*, *τ*)}, for any *T* ∈ *ℝ*, there exists a family of sets {*ℳ*(*t*)∣*t* ≤ *T*} satisfying the following:

(i) *ℳ*(*t*) is compact.
(ii) *ℳ*(*t*) is positively invariant; that is, *U*(*t*, *τ*)*ℳ*(*τ*) ⊂ *ℳ*(*t*).
(iii) ∀*t* ∈ (−*∞*, *T*], there exist *k*, *l* > 0 such that dist⁡(*U*(*t*, *τ*)*B*(*τ*), *ℳ*(*t*)) ≤ *ke* ^−(*t*−*τ*)^, that is, *ℳ*(*t*) pullback exponential attracts *B*(*τ*) for all {*B*(*t*)} ∈ *𝒟*.

Compared with the pullback exponential attractor, the fractal dimension of the weak *𝒟*-pullback exponential attractor is not necessarily uniformly bounded or even unbounded, and the positively invariant only holds for any *t* ∈ (−*∞*, *T*], compared with the *𝒟*-pullback attractor, which pullback attracts bounded set with exponential speed and contains *𝒟*-pullback attractor.

The paper is organized as follows. In Section 2, we recall some basic concepts about pullback attractor. In Section 3, we construct a weak *𝒟*-pullback exponential attractor for nonautonomous dynamical systems and we provided a method to verify the existence of weak *𝒟*-pullback exponential attractor. In Section 4, we apply our result to prove the existence of weak *𝒟*-pullback exponential attractor for nonautonomous reaction diffusion system in *H*_0_^1^ with exponential growth of the external force.

## 2. Preliminaries

Let *X* be a complete metric space; let *B*(*X*) be the set of all bounded subsets of *X*; *𝒟* is a nonempty class of parameterised sets $\hat{𝒟}=\left\{D(t)|t\in \mathbb{R},D(t)\in B(X)\right\}$ or $\hat{𝒟}=\left\{D(n)|n\in \mathbb{Z},D(n)\in B(X)\right\}$; and a two-parameter family of mappings {*U*(*t*, *τ*)∣*t* ≥ *τ*} = {*U*(*t*, *τ*)∣*t* ≥ *τ*, *t*, *τ* ∈ *ℝ*} act on *X*, that is, *U*(*t*, *τ*) : *X* → *X*, ∀*t* ≥ *τ*.

**Definition 1 .**

A two-parameter family of mappings {*U*(*t*, *τ*)} is said to be a process in *X*, if

(1) *U*(*t*, *s*)*U*(*s*, *τ*) = *U*(*t*, *τ*), ∀*t* ≥ *s* ≥ *τ*,
(2) *U*(*τ*, *τ*) = Id is the identity operator, *τ* ∈ *ℝ*.

The pair (*U*(*t*, *τ*), *X*) is generally referred to as a nonautonomous dynamical system, and (*U*(*n*, *m*), *X*) (*n*, *m* ∈ *ℤ*) is called a nonautonomous discrete dynamical system generated by (*U*(*t*, *τ*), *X*). If *x* → *U*(*t*, *τ*)*x* is continuous in *X*, we say that the process is continuous process; if *U*(*t*, *τ*)*x*_*n*_⇀*U*(*t*, *τ*)*x* as *x*_*n*_ → *x*, we say that the process is the norm-to-weak continuous process. Obviously, continuous process is also a norm-to-weak continuous process.

**Definition 2 .**

A family of sets {*B*(*t*)∣*t* ∈ *ℝ*} ∈ *𝒟* is called *𝒟*-pullback bounded absorbing sets for the process {*U*(*t*, *τ*)} if, for any *t* ∈ *ℝ* and any bounded sets {*D*(*t*)∣*t* ∈ *ℝ*} ∈ *𝒟*, there exists *τ*_0_(*t*, *D*(*t*)) ≤ *t* such that *U*(*t*, *τ*)*D*(*τ*) ⊂ *B*(*t*) for all *τ* ≤ *τ*_0_.

**Definition 3 .**

The family *𝒜* = {*𝒜*(*t*)∣*t* ∈ *ℝ*} ⊂ *B*(*X*) is said to be a *𝒟*-pullback attractor for *U*(*t*, *τ*) if the following hold:

(1) *𝒜*(*t*) is compact for all *t* ∈ *ℝ*;
(2) *𝒜* is invariant; that is, *U*(*t*, *τ*)*𝒜*(*τ*) = *𝒜*(*t*)  ∀*t* ≥ *τ*;
(3) *𝒜* is *𝒟*-pullback attracting; that is, lim_*τ*→−*∞*_dist⁡ (*U*(*t*, *τ*)*B*(*τ*), *𝒜*(*t*)) = 0, ∀{*B*(*t*)} ∈ *𝒟*, and *t* ∈ *ℝ*;
(4) if {*C*(*t*)}_*t*∈*ℝ*_ is another family of closed attracting sets, then *𝒜*(*t*) ⊂ *C*(*t*)  ∀*t* ∈ *ℝ*.

Here dist⁡(·, ·) denotes the nonsymmetric Hausdorff distance between sets in *X*; that is, dist⁡(*A*, *B*) = sup_*a*∈*A*_⁡inf_*b*∈*B*_⁡‖*a* − *b*‖.

**Definition 4 .**

The Kuratowski measure of noncompactness *α*(*B*) of *B* ⊂ *X* is defined by

(1) αB=inf⁡δ>0 ∣ B  admits  a  finite  cover  by  sets  of  diameter≤δ.

The following summarizes some of the basic properties of the measure of noncompactness.

**Lemma 5 (see [10]).**

Let *B*, *B*_1_, *B*_2_ ⊂ *X*. Then

(1) *α*(*B*) = 0 if, and only if, $\bar{B}$ is compact;
(2) *α*(*B*_1_ + *B*_2_) ≤ *α*(*B*_1_) + *α*(*B*_2_);
(3) *α*(*B*_1_) ≤ *α*(*B*_2_) for *B*_1_ ⊂ *B*_2_;
(4) *α*(*B*_1_ ∪ *B*_2_) ≤ max{*α*(*B*_1_), *α*(*B*_2_)};
(5) if *F*_1_⊃*F*_2_… are nonempty closed sets in *X* such that *α*(*F*_*n*_) → 0 as *n* → *∞*, then *F* = ⋂_*n*=1_^*∞*^ *F*_*n*_ is nonempty and compact.

In addition, let *X* be an infinite dimensional Banach space with a decomposition *X* = *X*_1_ ⊕ *X*_2_ and let *P* : *X* → *X*_1_, *Q* : *X* → *X*_2_ be projectors with dim⁡*X*_1_ < *∞*. Then

(6) *α*(*B*(*ε*)) = 2*ε*, where *B*(*ε*) is a ball of radius *ε*;
(7) *α*(*B*) < *ε* for any bounded subset *B* of *X* for which the diameter of *QB* is less than *ε*.

**Definition 6 (see [3–5]).**

A process {*U*(*t*, *τ*)} is called *𝒟*-pullback *ω*-limit compact for {*B*(*t*)∣*t* ∈ *ℝ*} if, for any *ε* > 0, there exists a *τ*_0_(*t*, *B*(*t*), *ε*) ≤ *t* such that *α*(⋃_*τ*≤*τ*_0__ *U*(*t*, *τ*)*B*(*τ*)) ≤ *ε*.

**Lemma 7 (see [3–5]).**

Assume that the process {*U*(*t*, *τ*)∣*t* ≥ *τ*} is *𝒟*-pullback *ω*-limit compact for $\hat{B}=\left\{B(t)|t\in \mathbb{R}\right\}$; then, for any sequence {*τ*_*n*_} ⊂ (−*∞*, *t*], *τ*_*n*_ → −*∞* as *n* → +*∞* and for any sequence *x*_*n*_ ∈ *B*(*τ*_*n*_), there exists a convergence subsequence of {*U*(*t*, *τ*_*n*_)*x*_*n*_} whose limit lies in $\omega (\hat{B},t)$; here $\omega (\hat{B},t)$ is defined by

(2) $$\begin{array}{c} \omega \left(\;\hat{B},t\;\right)=\underset{s\leq t}{⋂}\;\bar{\underset{\tau \leq s}{⋃}U\left(t,\tau \right)B\left(\tau \right)}. \end{array}$$

**Theorem 8 (see [3, 5]).**

Let {*U*(*t*, *τ*)∣*t* ≥ *τ*} be a continuous or norm-to-weak continuous process and {*U*(*t*, *τ*)∣*t* ≥ *τ*} is *𝒟*-pullback *ω*-limit compact; let {*B*(*t*)∣*t* ∈ *ℝ*} ⊂ *B*(*X*) be a family of *𝒟*-pullback bounded absorbing sets for the process. Then the process {*U*(*t*, *τ*)∣*t* ≥ *τ*} has a *𝒟*-pullback attractor *𝒜* = {*𝒜*(*t*)∣*t* ∈ *ℝ*}, and

(3) At⋂s≤t ⋃τ≤sUt,τBτ¯=⋂s≤t ⋃τ≤sUt,τB ∣ B∈BX¯.

For a discrete process {*U*(*n*, *m*)∣*n*, *m* ∈ *ℤ*, *n* ≥ *m*}, the above conclusions also hold true.

## 3. The Existence of Weak *𝒟*-Pullback Exponential Attractor

Let *X* be a Banach space; ‖·‖ denotes the norm of *X*, *𝒟* is a nonempty class of parameterised sets $\hat{𝒟}=\left\{D(t)|t\in \mathbb{R}\right\}\subset B(X)$ or $\hat{𝒟}=\left\{D(n)|n\in \mathbb{Z}\right\}\subset B(X)$, and {*U*(*t*, *τ*)} is a continuous process on *X*.

Now, we give our main theorems which describe the relationship between the measure of noncompactness and the weak *𝒟*-pullback exponential attractor.

**Theorem 9 .**

Assume that {*B*(*n*)} ∈ *𝒟* is positively invariant *𝒟*-pullback bounded absorbing sets of {*U*(*n*, *m*)}; that is, for any {*D*(*n*)} ∈ *𝒟*, *N* ∈ *ℤ*, there exists *T* ∈ *ℕ*, such that *U*(*n*, *m*)*D*(*m*) ⊂ *B*(*n*) for any *n* − *m* ≥ *T*, and *U*(*n*, *m*)*B*(*m*) ⊂ *B*(*n*) for any *m* ≤ *n* ≤ *N*; then the following are equivalent:

(I) The measure of noncompactness *𝒟*-pullback decays exponentially for the discrete process {*U*(*n*, *m*)}; that is, there exist *k*, *l* > 0 such that
  (4) α⋃k≤mUn,kBk≤ke−ln−m,for  any  m≤n≤N.
(II) The process {*U*(*n*, *m*)} has a weak *𝒟*-pullback exponential attractor; that is, there exists a family of sets {*ℳ*(*n*)∣*n* ≤ *N*} satisfying the following:
  (1) *ℳ*(*n*) is compact;
  (2) *ℳ*(*n*) is positively invariant; that is, *U*(*n*, *m*)*ℳ*(*m*) ⊂ *ℳ*(*n*);
  (3) {*ℳ*(*n*)∣*n* ∈ *ℤ*} attracts {*D*(*n*)} exponentially in a *𝒟*-pullback sense; more precisely,
    (5) dist⁡Un,mDm,Mn≤ke−ln−m,for  any  Dn∈D.

**Proof**

((I)⇒(II)) Since the measure of noncompactness *𝒟*-pullback decays exponentially for {*U*(*n*, *m*)}, from Definition 6, we find that {*U*(*n*, *m*)} is *𝒟*-pullback *ω*-limit compact. By Theorem 8, we get that

(6) $$\begin{array}{c} A\left(\;n\;\right)=\underset{k\leq n}{⋂}\;\bar{\underset{m\leq k}{⋃}U\left(n,m\right)B\left(m\right)} \end{array}$$

is a *𝒟*-pullback attractor of {*U*(*n*, *m*)}. Using (3) of Lemma 5, we find that

(7) αUn,mBmα⋃k≤mUn,kBk≤ke−ln−m,

and by the definition of the measure of noncompactness, for any *n* ≥ *m*, there exist finite points *x*_*n*,*i*_^*m*^ ∈ *B*(*n*) such that *U*(*n*, *m*)*B*(*m*) ⊂ ⋃_*i*=1_^*n*_*m*_^ *B*(*x*_*n*,*i*_^*m*^, *ke* ^−*l*(*n* − *m*)^). Letting *W*_*n*_^*m*^ = {*x*_*n*,*i*_^*m*^∣*i* = 1,2,…, *n*_*m*_} and *M*(*k*) = ⋃_*n*=0_^+*∞*^⋃_*i*=0_^*n*^ *U*(*k*, *k* − *i*)*W*_*k*−*i*_^*n*−*i*^, we get

(8) Mk+1=⋃n=0+∞ ⋃i=0nUk+1,k+1−iWk+1−in−i=⋃n=0+∞ ⋃i=0nUk+1,kUk,k+1−iWk+1−in−i⊃⋃n=1+∞ ⋃i=0nUk+1,kUk,k−i−1Wk−i−1n−1−i−1⊃Uk+1,k⋃n=1+∞ ⋃i=1nUk,k−i−1Wk−i−1n−1−i−1=Uk+1,k⋃n=1+∞ ⋃i=0n−1Uk,k−iWk−in−1−i=Uk+1,k⋃n=0+∞ ⋃i=0nUk,k−iWk−in−i=Uk+1,kMk.

Consequently, for all *n* ∈ *ℤ*, the family {*M*(*n*)∣*n* ≤ *N*} is positively invariant.

Let *ℳ*(*n*) = *M*(*n*) ∪ *𝒜*(*n*); we claim that {*ℳ*(*n*)∣*n* ≤ *N*} satisfies (II).

(Compactness) for any sequence *x*_*k*_ ∈ *ℳ*(*n*), there exist *m*_*k*_ and *y*_*k*_ such that *x*_*k*_ = *U*(*n*, *m*_*k*_)*y*_*k*_. By (I), we get that the process {*U*(*n*, *m*)} is pullback *𝒟*-*ω*-limit compact; we deduce from Lemma 7 that *x*_*k*_ has subsequence convergent in *ℳ*(*n*). We get that *ℳ*(*n*) is compact.

(Positively invariant) since *U*(*n* + 1, *n*)*M*(*n*) ⊂ *M*(*n* + 1), *U*(*n* + 1, *n*)*𝒜*(*n*) = *𝒜*(*n* + 1), we get

(9) Un+1,nMnUn+1,nMn∪An⊂Mn+1.

(Exponential attracting) for any {*D*(*m*)} ∈ *𝒟*, there exists *T* ∈ *ℕ*, such that

(10) $$\begin{array}{c} U\left(\;m,m-T\;\right)D\left(\;m-T\;\right)\subset B\left(\;m\;\right). \end{array}$$

Since {*B*(*n*)} is positively invariant, we get

(11) Un,m−TDm−T=Un,mUm,m−TDm−T⊂Un,mBm⊂⋃i=1nmBxn,im,ke−ln−m,

so we obtain

(12) dist⁡Un,m−TDm−T,Mn≤dist⁡Un,mBm,Mn.

Since *W*_*n*_^*m*^ ⊂ *M*(*n*), we get

(13)  dist⁡Un,m−TDm−T,Mn≤dist⁡Un,mBm,Wnm≤ke−ln−m

for any *n* ≥ *m*.

((II)⇒(I)) By the definition of dist⁡(·, ·), we get

(14) dist⁡Un,mBm,Mn=supx∈Bm⁡ infy∈Mn⁡dUn,mx,y≤ke−ln−m,

and, for any *x* ∈ *B*(*m*), we have

(15) $$\begin{array}{c} \underset{y\in M\left(n\right)}{\inf}d\left(\;U\left(\;n,m\;\right)x,y\;\right)\leq ke^{-l\left(n-m\right)}. \end{array}$$

Therefore, for any *x* ∈ *B*(*m*), there exists *y*_*x*_ ∈ *ℳ*(*n*), such that

(16) $$\begin{array}{c} d\left(\;U\left(\;n,m\;\right)x,y_{x}\;\right)<2ke^{-l\left(n-m\right)}. \end{array}$$

We get

(17) $$\begin{array}{c} U\left(\;n,m\;\right)x\in B\left(\;y_{x},2ke^{-l\left(n-m\right)}\;\right). \end{array}$$

Since *ℳ*(*n*) is a compact set, we get that there exist *y*_1_, *y*_2_,…, *y*_*l*_ ∈ *ℳ*(*n*) such that

(18) $$\begin{array}{c} M\left(\;n\;\right)\subset \overset{l}{\underset{i=1}{⋃}}B\left(\;y_{i},2ke^{-l\left(n-m\right)}\;\right). \end{array}$$

Therefore, for any *y*_*x*_, there exists *y*_*i*_*x*__ ∈ {*y*_1_, *y*_2_,…, *y*_*l*_} such that

(19) dyx,yix2ke−ln−m,dUn,mx,yixdUn,mx,yx+dyx,yix≤4ke−ln−m.

We get

(20) $$\begin{array}{c} U\left(\;n,m\;\right)B\left(\;m\;\right)\subset \overset{l}{\underset{i=1}{⋃}}B\left(\;y_{i},4ke^{-l\left(n-m\right)}\;\right), \end{array}$$

and, by Definition 4, we obtain

(21) $$\begin{array}{c} \alpha \left(\;U\left(\;n,m\;\right)B\left(\;m\;\right)\;\right)\leq Ke^{-l\left(n-m\right)}\;\left(K=4k\right), \end{array}$$

and, by (4) of Lemma 5, we get

(22) $$\begin{array}{c} \alpha \left(\;\overset{}{\underset{k\leq m}{⋃}}U\left(\;n,m\;\right)B\left(\;m\;\right)\;\right)\leq Ke^{-l\left(n-m\right)}, \end{array}$$

which say that the measure of noncompactness *𝒟*-pullback decays exponentially.

**Theorem 10 .**

Assume that {*B*(*t*)} ∈ *𝒟* is positively invariant *𝒟*-pullback bounded absorbing sets of {*U*(*t*, *τ*)}; that is, for any {*D*(*t*)} ∈ *𝒟*, *R* ∈ *ℝ*, there exists *T* ≥ 0, such that *U*(*t*, *τ*)*D*(*τ*) ⊂ *B*(*t*) for any *t* − *τ* ≥ *T*, and *U*(*t*, *τ*)*B*(*τ*) ⊂ *B*(*t*) for any *t* ≤ *R*, and there exists a continuous function *r*(*t*) that satisfies ‖*U*(*t*, *τ*)*x* − *U*(*t*, *τ*)*y*‖ ≤ *r*(*t*)‖*x* − *y*‖ for any *x*, *y* ∈ *B*(*τ*), *t* − *τ* ≤ 1; then the following are equivalent:

(I) The measure of noncompactness *𝒟*-pullback decays exponentially for the process {*U*(*t*, *τ*)}; that is, there exist *k*, *l* > 0 such that
  (23) α⋃s≤τUt,sBs≤ke−lt−τ,for  any  τ≤t≤R.
  (II) The process {*U*(*t*, *τ*)} has a weak *𝒟*-pullback attractor; that is, there exists a family of sets {*ℳ*(*t*)∣*t* ≤ *R*} satisfying the following:
  (1) *ℳ*(*t*) is compact;
  (2) *ℳ*(*t*) is positively invariant; that is, *U*(*t*, *τ*)*ℳ*(*τ*) ⊂ *𝒜*(*t*);
  (3) {*ℳ*(*t*)∣*t* ≤ *R*} attracts {*D*(*t*)} exponentially in *𝒟*-pullback sense; more precisely,
    (24) dist⁡Ut,τDτ,Mt≤kte−lt−τ,for  any  Dt∈D.

**Proof**

((I)⇒(II)) By Theorem 9, we know that the discrete process {*U*(*n*, *m*)} generated by {*U*(*t*, *τ*)} has a weak *𝒟*-pullback exponential attractor {*ℳ*(*n*)}, that is, *ℳ*(*n*) is compact and positively invariant and *𝒟*-pullback exponentially attracts {*D*(*n*)} ∈ *𝒟*. We set *ℳ*(*t*) = *U*(*t*, *k*)*ℳ*(*k*), *t* ∈ [*k*, *k* + 1), for all *k* ≤ *R*. As proof of Theorem 9, it is easy to prove that *ℳ*(*t*) is compact and positively invariant. Next, we will prove that *ℳ*(*t*) attracts {*D*(*n*)} ∈ *𝒟* exponentially in *𝒟*-pullback sense.

For any {*D*(*t*)} ∈ *𝒟*, there exists *T* ∈ *ℕ* such that *U*(*t*, *τ*)*D*(*τ*) ⊂ *B*(*t*) for any *t* − *τ* ≥ *T*. For discrete process {*U*(*n*, *m*)}, by Theorem 9, there exist *k*_0_, *l*_0_ > 0 such that

(25) $$\begin{array}{c} \mathrm{dist}\left(\;U\left(\;n,m\;\right)B\left(\;m\;\right),M\left(\;n\;\right)\;\right)\leq k_{0}e^{-l_{0}\left(n-m\right)}. \end{array}$$

For any *t*, *τ* ∈ *ℝ*, there exist *t*_0_, *τ*_0_ ∈ [0,1) such that *t* = *n* + *t*_0_, *τ* = *m* + *τ*_0_; therefore

(26) dist⁡Ut,τDτ,Mtdist⁡Un+t0,m+τ0Dm+τ0,Mn+t0≤dist⁡Un+t0,nUn,m+τ0Dm+τ0,Un+t0Mn≤rtdist⁡Un,m+τ0Dm+τ0,Mn≤rtdist⁡Un,m+T+1Um+T+1,m+τ0Dm+τ0,Mn≤rtdist⁡Un,m+T+1Bm+T+1,Mn≤rtk0e−l0n−m−T−1,dist⁡Ut,τDτ,Mtrtk0e−l0t−t0−τ+τ0−T−1≤rtk0e−l0−t0+τ0−T−1e−l0t−τ≤rtk0el0T+2e−l0t−τ.

We obtain that {*ℳ*(*t*)∣*t* ≤ *R*} attracts {*D*(*t*)} exponentially in a *𝒟*-pullback sense.

((II)⇒(I)) The proof is the same as that of Theorem 9, so we omit it.

We now present a method to verify that the measure of noncompactness *𝒟*-pullback decays exponentially for the process {*U*(*t*, *τ*)}.

Let *X* be a uniformly convex Banach space; that is, for all *ε* > 0, there exists *δ* > 0 such that, given *x*, *y* ∈ *X*, ‖*x*‖ ≤ 1, ‖*y*‖ ≤ 1, ‖*x* − *y*‖ > *ε*; then ‖*x* + *y*‖/2 < 1 − *δ*. Requiring a space to be uniformly convex is not a severe restriction in application, since this property is satisfied by all Hilbert spaces, the *L* ^*p*^ space with 1 < *p* < *∞*, and most Sobolev spaces *W* ^*k*,*p*^ with 1 < *p* < *∞*.

**Definition 11 (enhanced flattening property).**

Let *X* be a uniformly convex Banach space; for a family of bounded sets {*B*(*t*)} ⊂ *X*, there exist *k*, *l*, *T* > 0, and for any finite dimension subspace *X*_1_ of *X*, such that

(i) *P*_*m*_(⋃_*t*−*τ*≥*s*_ *U*(*t*, *τ*)*B*(*τ*)) is bounded;
(ii) ‖(*I* − *P*_*m*_)⋃_*t*−*τ*≥*s*_ *U*(*t*, *τ*)*x*‖ ≤ *ke* ^−*l*(*t*−*τ*)^ + *k*(*t*, *m*), ∀*x* ∈ *B*(*τ*),

for all *s* ≥ *T*. Here ‖·‖ denote the norm in *X* and *k*(*t*, *s*) is real-valued function satisfying

(27) $$\begin{array}{c} \underset{s\to +\infty }{\lim}k\left(\;t,s\;\right)=0. \end{array}$$

**Theorem 12 .**

Assume that the process {*U*(*t*, *τ*)} satisfies the enhanced flattening property; then the measure of noncompactness *𝒟*-pullback decays exponentially for {*U*(*t*, *τ*)}.

**Proof**

For any {*B*(*t*)} ∈ *𝒟*, from (2) and (7) of Lemma 5, and the enhanced flattening property, we get

(28) α⋃t−τ≥TUt,τBτ≤αPm⋃t−τ≥TUt,τBτ+αI−Pm⋃t−τ≥TUt,τBτ=αI−Pm⋃t−τ≥TUt,τBτ≤ke−lt−τ+kt,m.

Since *k*(*t*, *m*) → 0, for *ε*_0_ = *ke* ^−*l*(*t*−*τ*)^, there exists *M* > 0, for any *m* > *M*; we have

(29) $$\begin{array}{c} \left|\;k\left(\;t,m\;\right)\;\right|<ke^{-l\left(t-\tau \right)}. \end{array}$$

Hence, *α*(⋃_*t*−*τ*≥*T*_ *U*(*t*, *τ*)*B*(*t*)) ≤ 2*ke* ^−*l*(*t*−*τ*)^; that is, the measure of noncompactness of {*U*(*t*, *τ*)}*𝒟*-pullback decays exponentially.

Let *ℛ* be the set of all functions *r*(*t*) : *R* → (0, +*∞*) such that lim_*t*→−*∞*_ *t* ^*β*^ *e* ^*λt*^ *r* ^2^(*t*) = 0 for some *β* ≥ 0, *λ* > 0, and denote by *𝒟* the class of all families *𝒟* = {*D*(*t*)∣*t* ∈ *ℝ*} ⊂ *B*(*X*) such that $D(t)\subset \bar{B}(r(t))$ for some *r*(*t*) ∈ *ℛ*, $\bar{B}(r(t))$ denote the closed ball in *X* with radius *r*(*t*).

**Theorem 13 .**

Assume that the process {*U*(*t*, *τ*)} satisfies

(30) Ut,τuτ2≤K0t−τβe−λt−τuτ2+K1+K2eαt

for some *β* ≥ 0, 0 < *α* < *λ*, and *t* − *τ* ≥ *T*′ and for any *t* ≤ *R*; then the process {*U*(*t*, *τ*)} has a family of positively invariant *𝒟*-pullback bounded absorbing sets {*B*(*t*)∣*t* ≤ *R*}; that is, for any *D* ∈ *𝒟*, there exists *T* > 0 such that *U*(*t*, *τ*)*D*(*τ*) ⊂ *B*(*t*) for any *t* − *τ* ≥ *T* and *U*(*t*, *τ*)*B*(*τ*) ⊂ *B*(*t*).

**Proof**

Let us define

(31) $$\begin{array}{c} D\left(\;t\;\right)=\left\{\;x\in X\;|\;\left\|\;x\;\right\|\leq 2K_{1}+K_{2}e^{\alpha \left|t\right|}\;\right\},\;t\leq R. \end{array}$$

For every {*D*_0_(*t*)} ∈ *𝒟*, there exists *T*_0_ > 0 such that

(32) $$\begin{array}{c} U\left(\;t,t-s\;\right)D_{0}\left(\;t-s\;\right)\subset D\left(\;t\;\right),\;s\geq T_{0},\;t\leq R. \end{array}$$

Obviously, {*D*(*t*)} is a family of *𝒟*-pullback bounded absorbing sets. Moreover, there exists *T* > 0 such that

(33) $$\begin{array}{c} U\left(\;t,t-s\;\right)D\left(\;t-s\;\right)\subset D\left(\;t\;\right),\;s\geq T,\;t\leq R. \end{array}$$

Note that these can not hold for any *t* ∈ *ℝ*. Let

(34) $$\begin{array}{c} B\left(\;t\;\right)=\bar{\overset{}{\underset{s\geq T}{⋃}}U\left(t,t-s\right)D\left(t-s\right)},\;t\leq R. \end{array}$$

We know that *B*(*t*) ⊂ *D*(*t*) and {*B*(*t*)} is also a family of *𝒟*-pullback bounded absorbing sets. We also have

(35) Ut,τBτ=⋃s≥TUt,t−t−τ+sDt−t−τ+s¯⊂⋃s≥TUt,t−sDt−s¯=Bt.

By Theorems 10–13, we get the following theorem.

**Theorem 14 .**

Let *X* be a uniformly convex Banach space; {*U*(*t*, *τ*)} is a process on *X*, and the process {*U*(*t*, *τ*)} satisfies the following:

(I) ‖*U*(*t*, *τ*)*u*_*τ*_‖^2^ ≤ *K*_0_(*t* − *τ*)^*β*^ *e* ^−*λ*(*t*−*τ*)^‖*u*_*τ*_‖^2^ + *K*_1_ + *K*_2_ *e* ^*α*|*t*|^ for some *β* ≥ 0, 0 < *α* < *λ*, and *t* − *τ* ≥ *T* and any *t* ≤ *R*.
(II) ‖(*I* − *P*_*m*_)(⋃_*t*−*τ*≥*s*_ *U*(*t*, *τ*)*x*)‖ ≤ *ke* ^−*l*(*t*−*τ*)^ + *k*(*t*, *m*), ∀*x* ∈ *B*(*τ*) = {*x* : ‖*x*‖ ≤ 2*K*_1_ + *K*_2_ *e* ^*α*|*τ*|^}, for all *s* ≥ *T*. Here *m* is the dimension of subspace *X*_1_ of *X*, and *k*(*t*, *s*) is real-valued function that satisfies
  (36) $$\begin{array}{c} \underset{s\to +\infty }{\lim}k\left(\;t,s\;\right)=0. \end{array}$$
  (III) ‖*U*(*t*, *τ*)*x* − *U*(*t*, *τ*)*y*‖ ≤ *r*(*t*)‖*x* − *y*‖, for any *t* − *τ* < 1, *x*, *y* ∈ *B*(*τ*); then the process {*U*(*t*, *τ*)} has a weak *𝒟*-pullback exponential attractor; that is, for any *R* ∈ *ℝ*, there exists a family of sets {*ℳ*(*t*)∣*t* ≤ *R*} satisfying the following:
  (1) *ℳ*(*t*) is compact.
  (2) *ℳ*(*t*) is positively invariant; that is, *U*(*t*, *τ*)*ℳ*(*τ*) ⊂ *M*(*t*).
  (3) *ℳ*(*t*) attracts {*D*(*t*)} exponentially in a *𝒟*-pullback sense; more precisely,
    (37) dist⁡Ut,τDτ,Mt≤βte−lt−τ,for  any  Dt∈D.

## 4. Application to Nonautonomous Reaction Diffusion Equation

As an application of Theorem 14, we prove the existence of the weak *𝒟*-pullback exponential attractor in *H*_0_^1^(*Ω*) for the process generated by the solution of the following nonautonomous reaction diffusion equation:

(38) ut−▵u+fu=gt,x∈Ω,u∂Ω=0,uτ=uτ,

where *f* ∈ *C* ^1^(*ℝ*, *ℝ*), *g*(·) ∈ *L*_loc⁡_^2^(*ℝ*, *L* ^2^(*Ω*)), *Ω* is a bounded open subset of *ℝ* ^*n*^, and there exist *p* ≥ 2,  *c*_*i*_ > 0,  *i* = 1,…, 5, *l* > 0 such that
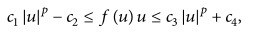
(39)

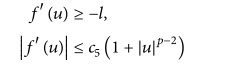
(40) for all *u* ∈ *ℝ*.

We set *A* : = −▵, naming *λ* the first eigenvalue of *A*, and denote *H* = *L* ^2^(*Ω*) by scalar product (·, ·) and norm |·|; let ((·, ·)) and ‖·‖ denote the scalar product and norm of *H*_0_^1^(*Ω*) and ((*u*, *v*)) = ∫_*Ω*_∇*u*∇*v* *dx* for all *u*, *v* ∈ *H*_0_^1^(*Ω*). Moreover, we suppose for any *t* ∈ *ℝ* that there exist *M* ≥ 0 and 0 ≤ *α* < *λ* such that

(41) $$\begin{array}{c} {\left|\;g\left(\;t\;\right)\;\right|}^{2}\leq Me^{\alpha \left|t\right|}. \end{array}$$

For this initial boundary value problem, we know from [7, 8] that, for any *τ*, *T* ∈ *ℝ*,  *T* > *τ*, there exists a unique solution *u*(·) ∈ *C*([*τ*, *T*]; *H*)∩*L* ^2^(*τ*, *T*; *H*_0_^1^(*Ω*))∩*L* ^*p*^(*τ*, *T*; *L* ^*p*^(*Ω*)).

Thanks to the existence theorem, the initial boundary value problem is equivalent to a process {*U*(*t*, *τ*)}_*t*≥*τ*_ defined by

(42) Ut,τ:H×τ,+∞⟶H01ut=Ut,τuτ,

where *u*(*t*) is the solution of (38)–(40) with *u*_*τ*_ as initial data at time *τ*.

**Theorem 15 (see [3]).**

Assume that *f* and *g* satisfy (39)–(41) and *u*(*t*) is a weak solution associated with (38). Then the following inequality holds for *t* > *τ*:

(43) ut2≤c1+t−τ+1t−τe−λt−τuτ2+1+1t−τ+1+1t−τe−λt∫−∞teλsgs2ds+1+1t−τe−λt∫−∞t∫−∞seλrgr2dr ds.

**Theorem 16 .**

Assume that *f* and *g* satisfy (39)–(41), where 2 ≤ *p* < +*∞*(*n* ≤ 2), 2 ≤ *p* ≤ (2*n* − 2)/(*n* − 2) (*n* ≥ 3). Then the process defined by (42) has a weak *𝒟*-pullback exponential attractor in *H*_0_^1^.

Next, we will prove that the process defined by (42) satisfy (I)–(III) of Theorem 14.

**Proof**

By (42), for *t* ≤ 0,

(44) e−λt∫−∞teλsgs2ds≤Me−λt∫−∞teλse−αsds≤Me−αtλ−α,

and, for *t* > 0,

(45) e−λt∫−∞teλsgs2ds=e−λt∫−∞0eλsgs2ds+∫0teλsgs2ds≤M1λ−α+eαtλ+α.

Therefore, for any *t* ∈ *ℝ*, we have

(46) $$\begin{array}{c} e^{-\lambda t}\overset{t}{\underset{-\infty }{\int }}e^{\lambda s}{\left|\;g\left(\;s\;\right)\;\right|}^{2}ds\leq \frac{M}{\lambda -\alpha }\left(\;1+e^{\alpha \left|t\right|}\;\right). \end{array}$$

Using the same proof, we can get

(47) $$\begin{array}{c} e^{-\lambda t}\overset{t}{\underset{-\infty }{\int }}\overset{s}{\underset{-\infty }{\int }}e^{\lambda r}{\left|\;g\left(\;r\;\right)\;\right|}^{2}dr\;ds\leq \frac{M}{{\left(\lambda -\alpha \right)}^{2}}\left(\;1+e^{\alpha \left|t\right|}\;\right). \end{array}$$

By (43) and using (46) and (47) we find that there exists *T* > 0, for any *t* − *τ* ≥ *T*; we have

(48) Ut,τuτ2≤K0t−τe−λt−τuτ2+K1+K2eαt.

By Theorem 13, for any fixed *R* ∈ *ℝ*, the process {*Ut*, *τ*}} generated by (38) is a family of positively invariant *𝒟*-pullback bounded absorbing sets {*B*(*t*)∣*t* ≤ *R*} and for any *x* ∈ *B*(*t*), ‖*x*‖ ≤ 2*K*_1_ + *K*_2_ *e* ^*α*|*t*|^.

Let *ℛ* be the set of all functions *r* : *R* → (0, +*∞*) such that lim_*t*→−*∞*_⁡*te* ^*λt*^ *r* ^2^(*t*) = 0 and denote by *𝒟* the class of all families *𝒟* = {*D*(*t*)∣*t* ∈ *R*} ⊂ *B*(*H*) such that $D(t)\subset \bar{B}(r(t))$ for some *r*(*t*) ∈ *ℛ*, $\bar{B}(r(t))$ denotes the closed ball in *H* with radius *r*(*t*).

Since *A* ^−1^ is a continuous compact operator in *H*, by the classical spectral theorem, there exist a sequence {*λ*_*j*_}_*j*=1_^*∞*^,

(49) 0<λ1≤λ2≤⋯≤λj≤⋯,λj⟶+∞,  as  j⟶∞,

and a family of elements {*e*_*j*_}_*j*=1_^*∞*^ of *H*_0_^1^(*Ω*) which are orthogonal in *H* such that

(50) $$\begin{array}{c} Ae_{j}=\lambda_{j}e_{j},\;\forall j\in N. \end{array}$$

Let *H*_*m*_ = span⁡{*e*_1_, *e*_2_,…, *e*_*m*_} in *H* and *P* : *H* → *H*_*m*_ is an orthogonal projector. For any *u* ∈ *H* we write

(51) $$\begin{array}{c} u=Pu+\left(\;I-P\;\right)u≜u_{1}+u_{2}. \end{array}$$

We set *u*_1_(*t*) = *U*(*t*, *τ*)*u*_1*τ*_ and *u*_2_(*t*) = *U*(*t*, *τ*)*u*_2*τ*_ to be solutions associated with (38) with initial data *u*_1*τ*_, *u*_2*τ*_ ∈ *B*(*τ*). Let *w*(*t*) = *u*_1_(*t*) − *u*_2_(*t*); by (38) we get

(52) $$\begin{array}{c} w_{t}-▵w+f\left(\;u_{1}\left(\;t\;\right)\;\right)-f\left(\;u_{2}\left(\;t\;\right)\;\right)=0. \end{array}$$

Taking inner product of (52) with −▵*w* in *H*, we have

(53) $$\begin{array}{c} \frac{1}{2}\frac{d}{dt}{\left\|\;w\;\right\|}^{2}+{\left|\;▵w\;\right|}^{2}+\left(\;f\left(\;u_{1}\;\right)-f\left(\;u_{2}\;\right),-▵w\;\right)=0. \end{array}$$

Taking into account (40) and Hölder inequality, it is immediate to see that

(54) fu1−fu2,−▵w≤∫Ωfu1−fu2▵wdx≤12▵w2+12·∫Ωfu1−fu22dx,∫Ωfu1−fu22dx=∫Ωf′u1+θu2−u12u1−u22dx≤c∫Ω1+u1p−2+u2p−22u1−u22dx≤c∫Ω1+u12p−1+u22p−1dxp−2/p−1·∫Ωu1−u22p−11/p−1≤c1+u12p−12p−2+u22p−12p−2w2p−12.

Since 2 ≤ *p* < *∞*(*n* ≤ 2), 2 ≤ *p* ≤ *n*/(*n* − 2) + 1(*n* ≥ 3), using Sobolev embedding theorem, we obtain

(55) ∫Ωfu1−fu22dx≤c1+u12p−2+u22p−2w2≤cw2.

Since *u*_1_(*t*), *u*_2_(*t*) ∈ *B*(*t*), we get

(56) $$\begin{array}{c} {\left\|\;u_{i}\left(\;t\;\right)\;\right\|}^{2}\leq 2K_{1}+K_{2}e^{\alpha \left|t\right|},\;i=1,2. \end{array}$$

From (53)–(56), we have

(57) $$\begin{array}{c} \frac{d}{dt}{\left\|\;w\;\right\|}^{2}\leq c\left(\;1+e^{\alpha \left|t\right|}\;\right){\left\|\;w\;\right\|}^{2}. \end{array}$$

Therefore

(58) $$\begin{array}{c} {\left\|\;w\left(\;t\;\right)\;\right\|}^{2}\leq e^{c\left(1+\int_{t-1}^{t}e^{\alpha \left|s\right|}ds\right)}{\left\|\;w\left(\;\tau \;\right)\;\right\|}^{2},\;t-\tau <1. \end{array}$$

We obtain

(59) Ut,τu1τ−Ut,τu2τ2≤ec1+∫t−1teαsdsu1τ−u2τ2.

For any *u* ∈ *H* we write

(60) $$\begin{array}{c} u=P_{m}u+\left(\;I-P_{m}\;\right)u≜u_{1}+u_{2}. \end{array}$$

Taking the inner product of (38) with −▵*u*_2_, we have

(61) 12ddtu22+▵u22+fu,−▵u2=gt,−▵u2.

Applying the Poincaré inequality and Hölder inequality, we get

(62) $$\begin{array}{c} \frac{d}{dt}{\left\|\;u_{2}\;\right\|}^{2}+\lambda_{m}{\left\|\;u_{2}\;\right\|}^{2}\leq 2\left(\;\int_{\Omega }{\left|\;f\left(\;u\;\right)\;\right|}^{2}dx+{\left|\;g\left(\;t\;\right)\;\right|}^{2}\;\right). \end{array}$$

By (40), we find

(63) $$\begin{array}{c} \left|\;f\left(\;u\;\right)\;\right|\leq c\left(\;{\left|\;u\;\right|}^{p-1}+1\;\right). \end{array}$$

Hence,

(64) ddtu22+λmu22≤c1+∫Ωus2p−2dx+gt2.

Thanks to Sobolev embedding theorem, we obtain

(65) $$\begin{array}{c} \frac{d}{dt}{\left\|\;u_{2}\;\right\|}^{2}+\lambda_{m}{\left\|\;u_{2}\;\right\|}^{2}\leq c\left(\;1+{\left\|\;u\;\right\|}^{2p-2}+{\left|\;g\left(\;t\;\right)\;\right|}^{2}\;\right). \end{array}$$

Since *u*(*t*) ∈ *B*(*t*), hence

(66) $$\begin{array}{c} {\left\|\;u\;\right\|}^{2p-2}\leq c\left(\;1+e^{\alpha \left(p-1\right)\left|t\right|}\;\right). \end{array}$$

By (41), we get

(67) $$\begin{array}{c} \frac{d}{dt}{\left\|\;u_{2}\;\right\|}^{2}+\lambda_{m}{\left\|\;u_{2}\;\right\|}^{2}\leq c\left(\;1+e^{\alpha \left(p-1\right)\left|t\right|}+e^{\alpha \left|t\right|}\;\right). \end{array}$$

Using Gronwall lemma, we have

(68) u2t2≤e−λmt−τu2τ2+ce−λmt∫−∞teλms1+eαp−1s+eαsds≤ce−λm−λt−τe−λt−τuτ2+1λm+1λm−αp−1+1λm−α+eαp−1tλm−αp−1+eαtλm−α.

Since *e* ^−*λ*(*t*−*τ*)^‖*u*_*τ*_‖^2^ → 0 for any *u*_*τ*_ ∈ *B*(*τ*) and *λ*_*m*_ → +*∞*, which imply that there exists *T* > 0, for any *t* − *τ* ≥ *T*, we have

(69) $$\begin{array}{c} {\left\|\;u_{2}\left(\;t\;\right)\;\right\|}^{2}\leq c\left(\;e^{-\left(\lambda_{m}-\lambda \right)\left(t-\tau \right)}+k\left(\;t,m\;\right)\;\right). \end{array}$$

Here *k*(*t*, *m*) = 1/*λ*_*m*_ + 1/(*λ*_*m*_ − *α*(*p* − 1)) + 1/(*λ*_*m*_ − *α*) + *e* ^*α*(*p*−1)|*t*|^/(*λ*_*m*_ − *α*(*p* − 1)) + *e* ^*α*|*t*|^/(*λ*_*m*_ − *α*); obviously *k*(*t*, *m*) → 0 as *m* → +*∞*.

Therefore, by (48), (59), and (69), the process {*U*(*t*, *τ*)}_*t*≥*τ*_ generated by (38) satisfies all the conditions of Theorem 14.
